# The importance of combining serological testing with RT-PCR assays for efficient detection of COVID-19 and higher diagnostic accuracy

**DOI:** 10.7717/peerj.15024

**Published:** 2023-04-11

**Authors:** Sawsan S. Alamri, Ahdab Alsaieedi, Yousef Khouqeer, Marwah Afeef, Samiyah Alharbi, Abdullah Algaissi, Maimonah Alghanmi, Tarfa Altorki, Ayat Zawawi, Mohamed A. Alfaleh, Anwar M. Hashem, Rowa Alhabbab

**Affiliations:** 1 Vaccines and Immunotherapy Unit, King Fahd Medical Research Centre, King Abdulaziz University, Jeddah, Saudi Arabia; 2 Department of Medical Laboratory Sciences, Faculty of Applied Medical Sciences, King Abdulaziz University, Jeddah, Saudi Arabia; 3 College of Medicine, King Saud bin Abdulaziz University for Health Sciences, Jeddah, Saudi Arabia; 4 Study & Research Department, King Fahad General Hospital, Jeddah, Saudi Arabia; 5 Intensive Care Unit, King Fahad General Hospital, Jeddah, Saudi Arabia; 6 Department of Medical Laboratories Technology, College of Applied Medical Sciences, Jazan University, Jazan, Saudi Arabia; 7 Medical Research Centre, Jazan University, Jazan, Saudi Arabia; 8 Department of Pharmaceutics, Faculty of Pharmacy, King Abdulaziz University, Jeddah, Saudi Arabia; 9 Department of Medical Microbiology and Parasitology, Faculty of Medicine, King Abdulaziz University, Jeddah, Saudi Arabia

**Keywords:** SARS-CoV-2, Serology, COVID-19, Diagnostic, Testing profile, Infection, RT-PCR, Healthcare worker

## Abstract

Misdiagnosing suspected COVID-19 individuals could largely contribute to the viruses transmission, therefore, making an accurate diagnosis of infected subjects vital in minimizing and containing the disease. Although RT-PCR is the standard method in detecting COVID-19, it is associated with some limitations, including possible false negative results. Therefore, serological testing has been suggested as a complement assay to RT-PCR to support the diagnosis of acute infections. In this study, 15 out of 639 unvaccinated healthcare workers (HCWs) were tested negative for COVID-19 by RT-PCR and were found seropositive for SARS-CoV-2 nucleocapsid protein-specific IgM and IgG antibodies. These participants underwent additional confirmatory RT-PCR and SARS-CoV-2 spike-specific ELISA tests. Of the 15 individuals, nine participants were found negative by second RT-PCR but seropositive for anti-spike IgM and IgG antibodies and neutralizing antibodies confirming their acute infection. At the time of collection, these nine individuals were in close contact with COVID-19-confirmed patients, with 77.7% reporting COVID-19-related symptoms. These results indicate that including serological tests in the current testing profile can provide better outcomes and help contain the spread of the virus by increasing diagnostic accuracy to prevent future outbreaks rapidly.

## Introduction

The newly emerged virus, severe acute respiratory syndrome coronavirus-2 (SARS-CoV-2), has resulted in more than two years of global outbreaks of coronavirus disease 2019 (COVID-19) due to viral evolution. Several variants of SARS-CoV-2 have emerged with better viral fitness compared to their ancestral strains. This evolutionary progress is attributed to their increased receptor affinity, infectivity, viral replication, transmissibility and immune escape than the ancestral strain (Cherian et al., 2021; Dougherty et al., 2021; Farinholt et al., 2021b; Kumar et al., 2021). The first set of mutations is the alpha variant of concern (VOC), which resulted in a 50% higher transmissibility rate and more severe disease without any apparent effect on neutralization capacity by convalescent serum and vaccine-induced antibodies compared to its ancestral strain (Challen et al., 2021; Duong, 2021; Safaie et al., 2022; Supasa et al., 2021). Several other VOCs, such as Beta, Gamma, Delta, alongside others emerging between 2020 and 2021 displayed variable level of impact on transmissibility, severity and immunity (Bast et al., 2021; Betton et al., 2021; Cele et al., 2021; Duong, 2021; Louis et al., 2021; Madhi et al., 2021; Wall et al., 2021; Wang et al., 2021; Wibmer et al., 2021; Zhou et al., 2021). Since the emergence of the BA.1 Omicron VOC in late 2021, several other subvariant have been reported. However, the impact of Omicron mutations on disease severity and virus transmission rate is not fully elucidated, although some reports suggest an increase in transmission rate (Dougherty et al., 2021; Duong, 2021; Saxena et al., 2021; Teyssou et al., 2021) and less severity (Lorenzo-Redondo, Ozer & Hultquist, 2022; Nyberg et al., 2022).

Despite a large number of vaccinated individuals and the observed protective effect of immunization in attenuating disease severity, vaccinated people still could get infected and serve as a transmission source (Farinholt et al., 2021b; Rotondo et al., 2021; Subbarao, 2021; Tenforde et al., 2021). Therefore, it is crucial to differentiate infected from non-infected individuals, especially in healthcare facilities where maintaining functional patient care is essential. Although RT-PCR is the golden standard diagnostic method for viral RNA detection from upper respiratory tract swab samples, it can provide false-negative results. (Jacofsky, Jacofsky & Jacofsky, 2020; Udugama et al., 2020; Wolfel et al., 2020). Serological tests can significantly enhance the diagnostic efficiency. Individuals’ immunity can be measured by detecting their antibody reactivity—including IgM and IgG—either by using ELISA or *via* rapid lateral flow immunoassays. Moreover, serology can differentiate between coronaviruses, including SARS-CoV-1, SARS-CoV-2, and MERS-CoV, upon testing for receptor binding domain(RBD)- or spike-specific antibodies.

In this study, nine seropositive participants in close contact with COVID-19 confirmed cases and expressing COVID-19-related symptoms were found; however, tested negative twice by nasopharyngeal/oropharyngeal swab RT-PCR one day before and two days after their positive serological testing. Thus, we investigated the importance of combining serology testing with RT-PCR to increase COVID-19 diagnostic accuracy.

## Methods

### Sampling

This study extends a previous report published by our group (Alhabbab et al., 2021). Briefly, serum samples were collected randomly and cross-sectionally from 693 HCWs at three leading referral hospitals in Jeddah and those working at five COVID-19 quarantine sites. This study was conducted from 29 June 2020 to 1 April 2021 in non-vaccinated HCWs before the vaccine introduction. All participants were working at the designated hospitals and locations at the time of the epidemic outbreak, and they were all over 21 years old. Healthcare professionals collected all samples in yellow top tubes at the sites and stored them at 4 °C until transported to the laboratory for testing within a maximum of 3 h from collection. The results for SARS-CoV-2-nucleocapsid protein IgM and IgG-specific antibodies were released within 24 h of collection. The inclusion criteria included seropositivity for SARS-CoV-2-nucleocapsid and spike proteins binding IgM and IgG antibodies by ELISA, positivity for neutralizing antibodies and negative COVID-19 RT-PCR results before and after blood collection. More details are provided below in the results section.

All procedures and methods were performed following the relevant guidelines and regulations, including the ethical standards of the Helsinki Declaration of the World Medical Association. The study was conducted according to the ethical approval from the Institutional Review Board at the Ministry of Health (MOH), Saudi Arabia (IRB Numbers: H-02-J-002 and Project Number: 1367). Samples were anonymized, and all participants signed informed consent.

Participants were contacted and included in the study based on the inclusion and exclusion criteria described. All participants completed a survey including the following information: demographics (name, age, sex, contact details, *etc*.), symptoms experienced during blood collection, and in close contact with COVID-19 confirmed cases. Participants’ RT-PCR data was obtained from the system of the Ministry of Health.

### RT-PCR for COVID-19

This test was done in collaboration with the MOH. All samples were collected by well-trained healthcare personnel in viral transport media. The virus RNA was then extracted from the samples using virus mini kit v2.0 on the EZ1 Advanced XL instrument (Qiagen, Hilden, Germany), as instructed by the manufacturer. After obtaining 60 µl from the extracted RNA materials, the PCR-mix was prepared using BGI Kit according to the manufacturer’s instructions. Next, 10 µl from the extracted RNA samples was added to 20 µl from the prepared PCR-mix into the appropriate well (96-well plate). The plates were then centrifuged and placed into the Lightcycler 480 RT-PCR system (Roche, Basel, Switzerland), which was programmed as recommended by the manufacturer.

### ELISA (enzyme-linked immunosorbent assay) for SARS-CoV-2 antibodies detection

We used a validated in-house ELISA test to detect SARS-CoV-2 specific IgM and IgG antibodies in serum for the most immunogenic SARS-CoV-2 antigens, including nucleocapsid and spike proteins (Algaissi et al., 2020). Briefly, ELISA plates were coated with our in-house recombinant nucleocapsid protein or commercial recombinant spike-1 subunit protein (Sino biological, Beijing, China) overnight at 4 °C, as described previously. After washing, the plates were blocked, washed, and incubated with 1:100 diluted serum samples. Subsequently, the plates were washed and incubated with anti-human IgG or anti-human IgM conjugated to HRP. The plates were then washed and incubated with 3, 3′, 5, 5′-tetramethylbenzidine (TMB) substrate (KPL, Gaithersburg, MD, USA). The reaction was then stopped by 0.16 M sulfuric acid. ELx808 microplate reader (BioTek, Winooski, VT, USA) was used to measure the absorbance at 450 nm. The ELISA cut-off values were 0.4, 0.55, 0.17 and 0.3 for IgG nucleocapsid-ELISA, IgM N-ELISA, IgG spike-1-ELISA and IgM spike-1-ELISA, respectively, as previously calculated (Algaissi et al., 2020).

### Cells used for the neutralization assay

Cells used in this study included the African Green monkey kidney-derived Vero E6 cell line (1586; ATCC, Manassas, VA, USA) and Baby Hamster kidney BHK-21/WI-2 cell line (EH1011; Kerafast, Boston, MA, USA). Cells were maintained in complete Dulbecco’s modified essential medium (DMEM) supplemented with penicillin (100 U/mL), streptomycin (100 µg/mL) and 5 or 10% fetal bovine serum (FBS) in a 5% CO_2_ environment at 37 °C.

### rVSV- ΔG/SARS-2-S*-luciferase pseudovirus neutralization assay

The used neutralization assay pseudovirus based on the recombinant vesicular stomatitis virus (VSV) bearing SARS-CoV-2 spike protein (rVSV- ΔG/SARS-2-Spike*-luciferase pseudovirus), which was utilized as we have previously described (Almahboub et al., 2020).

### Statistical analysis

The chi-square test was used to determine the categorical variables’ association, while the *t*-test was used to compare two quantitative variables. Statistical analysis and graphical presentations were generated using GraphPad Prism version 9.0.2 software (Graph-Pad Software, Inc., CA, USA).

## Results

### Characteristics of the study applicants

**Figure 1:**
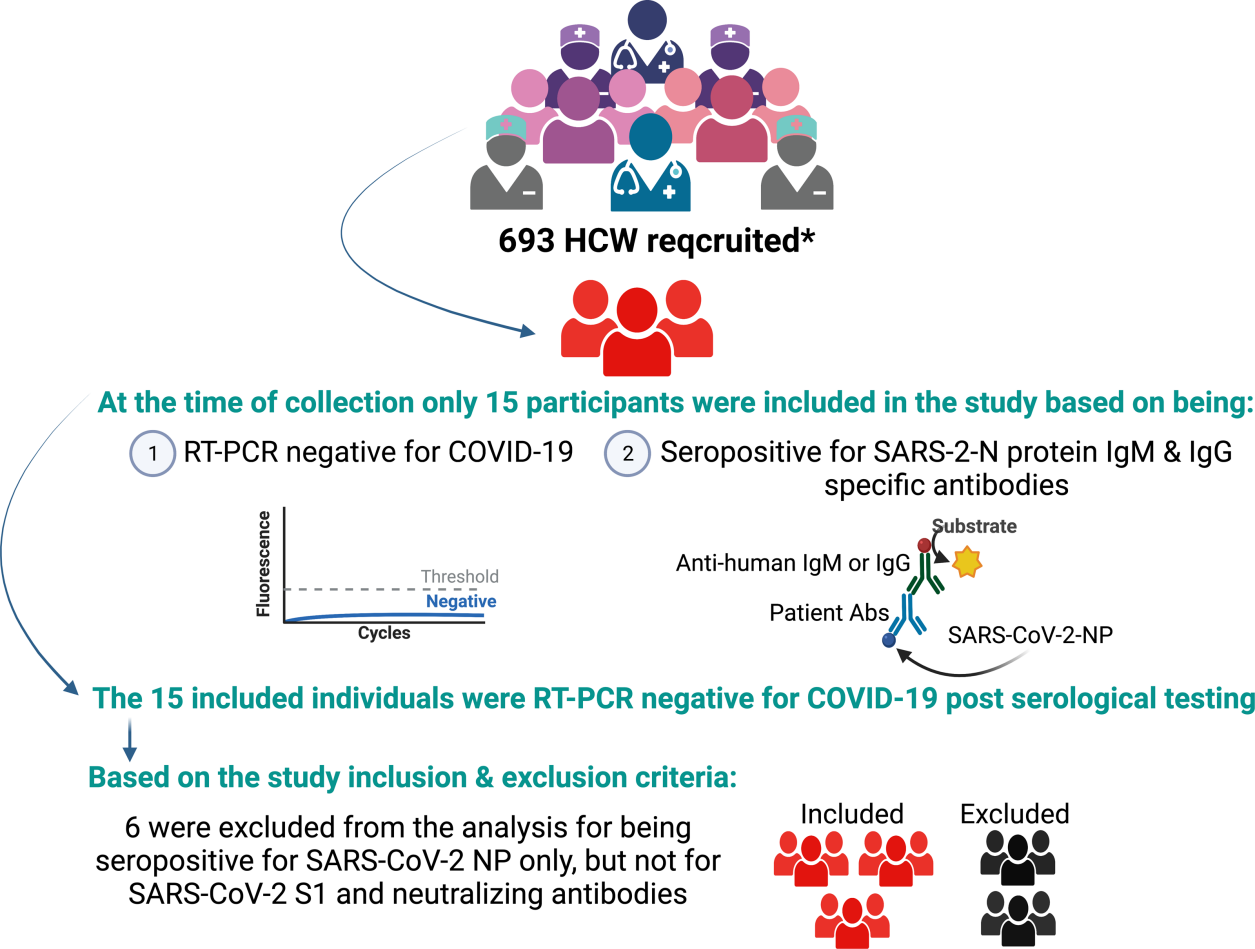
Study profile. The asterisk (*) in the figure refers to Alhabbab et al. (2021). Created with BioRender (https://www.biorender.com/).

Among 693 recruited HCWs, 15 participants had negative RT-PCR results for COVID-19 one day before their serological testing. However, they were found to be seropositive for IgM and IgG antibodies specific to the SARS-CoV-2 nucleocapsid protein. Two days later, they were tested again for COVID-19 by RT-PCR and found negative. To confirm the seropositivity of these individuals, we examined their serum samples for SARS-CoV-2 spike-1 protein-specific IgM- and IgG antibodies by ELISA. We found that 6 of the participants were seronegative for anti-spike protein antibodies as well as neutralizing antibodies. Therefore, they were excluded from the analysis (Fig. 1). The study population included one male (11.1%) and eight females (88.8%) HCWs. Only two (22.22%) were laboratory staff, while the remaining (77.7%) were medical personnel with direct patient contact. Moreover, none of the participants was previously diagnosed with or vaccinated against COVID-19 (Table S1).

### Dynamic changes in SARS-CoV-2 antibodies in RT-PCR negative HCWs

**Figure 2:**
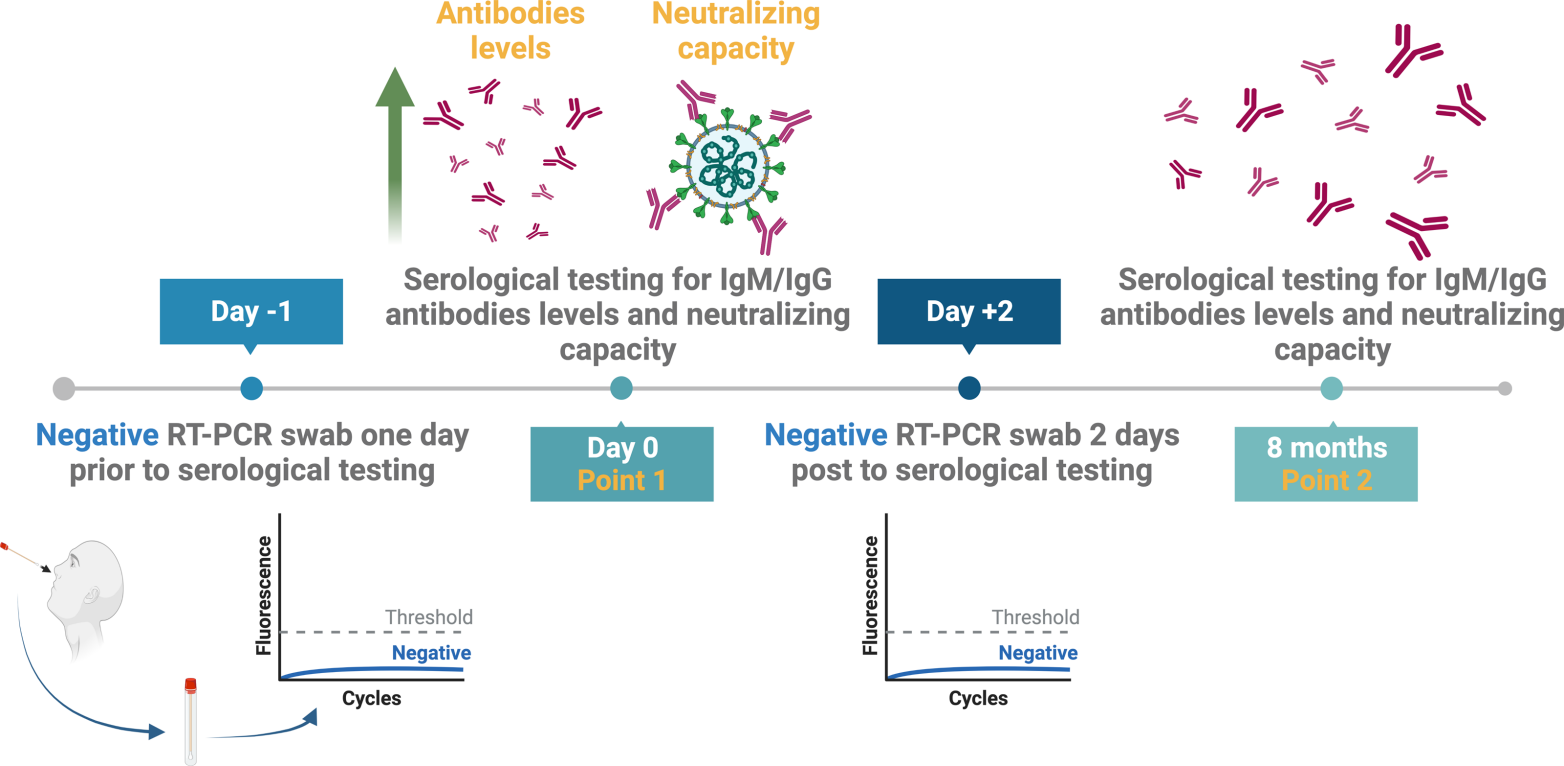
Study design and samples collection timeline. Created with BioRender.com; ©Biorender.

The results of the nine included individuals based on their seropositivity for SARS-CoV-2 nucleocapsid, spike-protein specific binding, and neutralizing antibodies were confirmed by serological testing eight months later (Fig. 2). Thus, new serum samples were collected from all participants after eight months to re-measure the levels of their antibodies by ELISA and to test for their neutralization capacity (Fig. 2).

**Figure 3:**
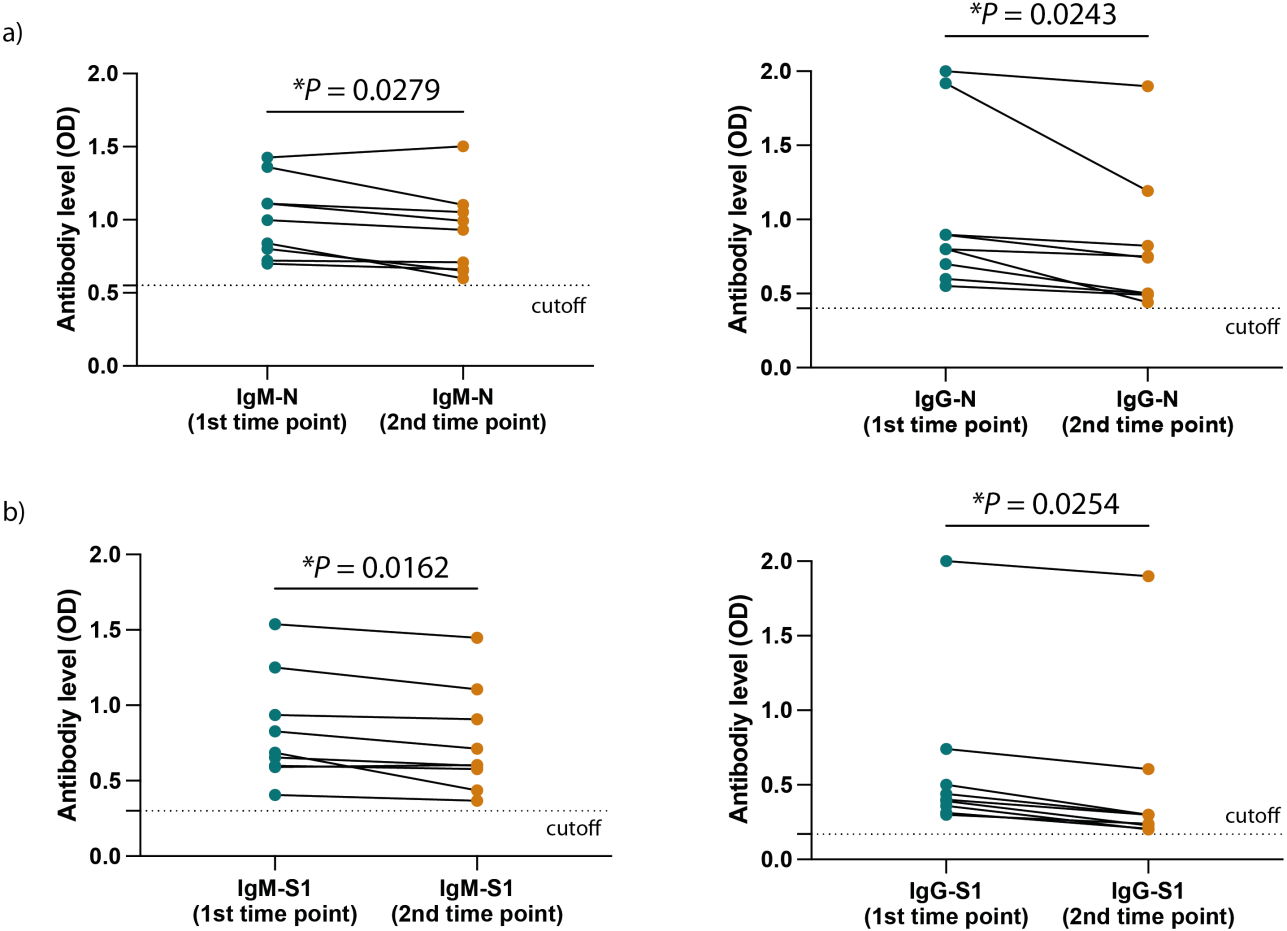
SARS-CoV-2 antibodies level in the HCWs included participants. (A) The levels of SARS-CoV-2 N protein specific IgM (left) and IgG (right) as well as (B) the levels of SARS-CoV-2 S1 protein specific IgM (left) and IgG (right) at the two different time points of collection. Statistics were calculated by paired *t*-test, ^*^P < 0.05.

**Figure 4:**
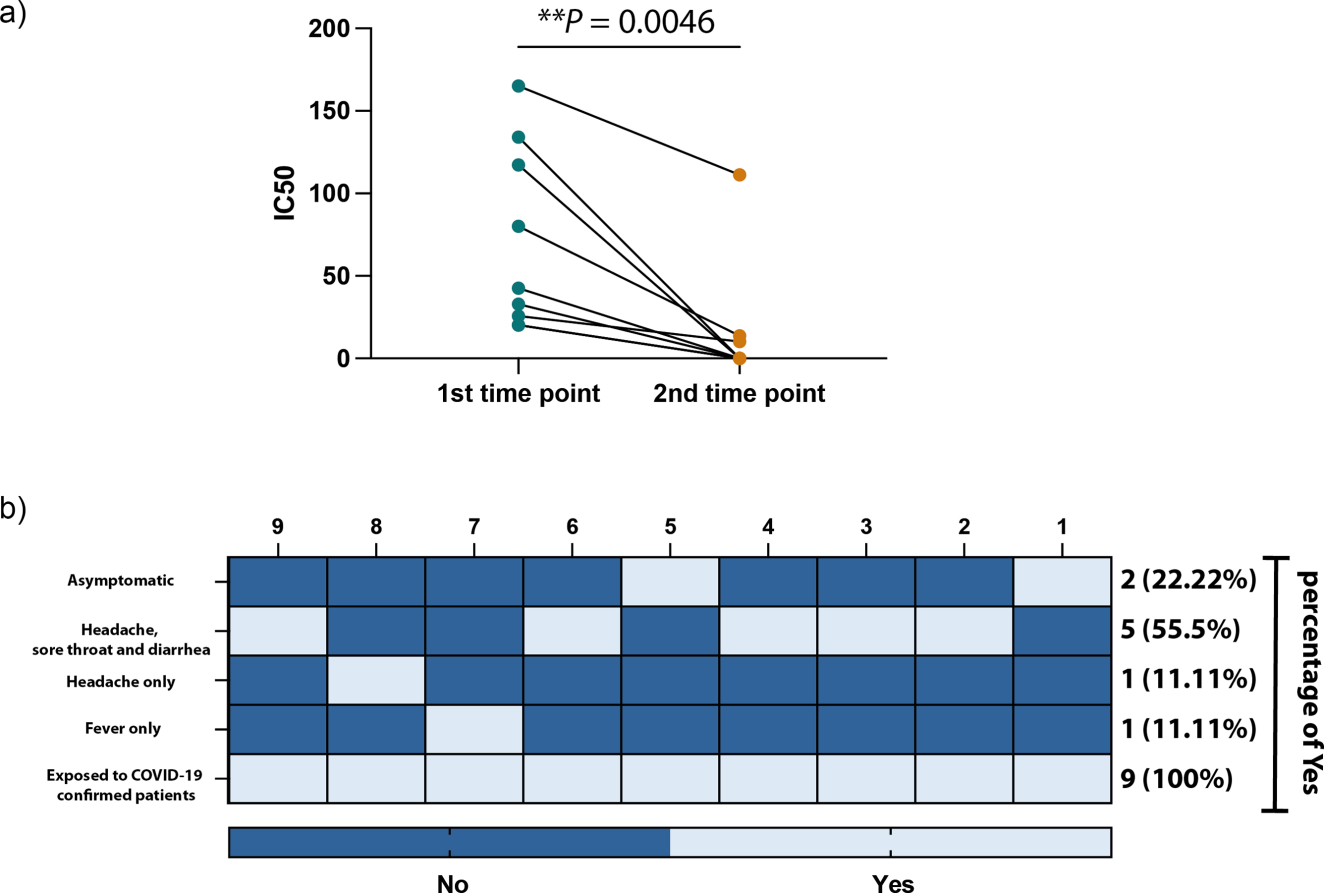
SARS-CoV-2 antibodies efficiency and symptoms associated with HCWs included participants. (A) IC50 values of SARS-CoV-2 neutralization antibodies against each collection time point in our study. (B) Heatmap illustrating the COVID-19 related symptoms expressed by each participant. Statistics were calculated by paired *t*-test, ^*^P < 0.05 & ^**^P < 0.005.

Figures 3A and 3B show a significant difference in the ELISA optical densities (ODs) results for SARS-CoV-2 nucleocapsid-and spike-1 IgM and IgG antibodies between the two-time points of collection was observed with a clear decline in the levels of these antibodies with time. Furthermore, sera from most individuals showed a significant reduction in neutralizing antibody titre over time, except for one participant who seemed to maintain high levels of SARS-CoV-2 neutralizing antibodies up to eight months following infection (Fig. 4A).

### Factors associated with seropositive as well as RT-PCR negative participants

All participants were RT-PCR negative twice, although they had direct exposure to COVID-19 confirmed cases, with 77.7% (7/9) of participants experiencing symptoms and 11% (2/9) being asymptomatic (Fig. 4B). The main COVID-19-related symptoms observed by participants were headaches combined with sore throat and diarrhea, and only a few subjects experienced headaches or fever.

As shown in Table S2, nasopharyngeal swabs were initially collected from five of the participants, while four undertook oropharyngeal swabs. The same collection method was used for consistency during the second collection, after two days from the initial point. Moreover, we have found that although the initial population of 693 participants consisted almost of an equal number of male (*n* = 346) and female (*n* = 347) participants, most of the subjects included here were females, with only one male (Table S2). Also, all participants were young HCWs in contact with COVID-19-confirmed patients (Tables S1 and S2).

## Discussion

Nowadays, challenges in managing and diagnosing SARS-CoV-2 infection are increasing due to the continuous evolution of the virus and the emergence of multiple variants (Safarchi et al., 2021; Zimmerman et al., 2021). Omicron VOC and its subvariants, so far, are the most commonly prevalent strains circulating globally with high transmission rates and the ability to evade vaccine-induced immunity compared to the previously reported variants (Dougherty et al., 2021; Farinholt et al., 2021a; Focosi et al., 2021; Kumar et al., 2021; Lesbon et al., 2021). The current RT-PCR or rapid antigen testing assays have been reported to be associated with false-negative results, which can be due to the timing and quality of the collected swab samples—especially during the declining phase of the viral load in the upper respiratory tract (Jacofsky, Jacofsky & Jacofsky, 2020; Wolfel et al., 2020). Therefore, obtaining false-negative RT-PCR for COVID-19 is not uncommon (Vengesai et al., 2021). A study collecting data from the fever clinic of Beijing Haidion Hospital showed that between every ten RT-PCR-negative cases, two were established to be true COVID-19-positive, yielding a rate of around 20% false-negative RT-PCR results (Li et al., 2020). Moreover, the virus has been detected in anal and blood swabs but not in oral swabs obtained from the same individuals diagnosed as COVID-19-negative (Zhang et al., 2020). These observations explain the nine seropositive cases of active infection in our study, expressing COVID-19-related symptoms and in close contact with confirmed cases, but were tested RT-PCR-negative twice at two different points.

Although the dynamic of COVID-19-specific antibodies is not well established, measuring serum-specific COVID-19-antibodies, which can be generated rapidly following infection, can serve as a highly sensitive and accurate aiding tool to compensate for the reported RT-PCR limitations (Bruni et al., 2020; Lauer et al., 2020; Vengesai et al., 2021). Serology has also been reported to be a more practical substitute for chest computed tomography (Guo et al., 2020; Lippi & Plebani, 2020; Qian et al., 2020). Here, all nine included participants had high antibody levels during the first collection time across the period of expressing COVID-19-related symptoms. Notably, these levels decreased after eight months. These data are consistent with previous studies reporting the persistence of circulating antibodies for up to a year post-recovery (Dan et al., 2021; Xiang et al., 2021). These results indicate that all nine individuals were seropositive for antibodies against viral antigens at both time points, confirming their previous exposure to SARS-CoV-2 and the importance of serology.

Most of the participants in our study expressed COVID-19-related symptoms, and they were all in close contact with confirmed COVID-19 cases. Moreover, they were all HCWs who had never been isolated and were more likely to spread the infection silently. Notably, all HCWs have continuous access to RT-PCR testing, and the MOH frequently tests them to minimize the spread of infection. Although the reported symptoms by the nine participants do not include the most predominant COVID-19-related symptoms, such as ageusia and anosmia, those symptoms vary among regions. For example, it has been reported that ageusia and anosmia are more frequent in Europe and US than in China and Saudi Arabia (Alhabbab et al., 2021; Wang et al., 2022). Therefore, due to the close contact of these nine participants to COVID-19 confirmed patients at the time of collection and the frequent RT-PCR testing they are exposed to, it is unusual that other infectious agents cause their symptoms. Additionally, despite the method used to collect the swab samples for RT-PCR, all participants in our study showed similar results, including negative RT-PCR and positive serology tests. These findings suggest that obtaining a false negative result in RT-PCR may not be affected by the type of the swab sample.

The limitation of our study was that it was performed on samples obtained during the outbreak of the ancestral SARS-CoV-2. Therefore, we could not include samples from subjects infected with the newly emerged SARS-CoV-2 variants nor from vaccinated individuals. However, the ancestral SARS-CoV-2 virus has a slower transmission rate than the newly emerged strains, and still, we could detect nine SARS-CoV-2 seropositive individuals with two negative RT-PCR results among HCWs. Alongside the fact that Delta and Omicron possess a higher transmission rate than the ancestral SARS-CoV-2 and might contribute to a higher rate of false-negative RT-PCR results, we expect high numbers of undocumented cases participating in the disease spread. Therefore, to overcome these issues, incorporating serological testing in the standard diagnostic methods to detect SARS-CoV-2, especially at the screening stage, is essential. An additional limitation is the small sample size included in this study; however, such cases can only be detected with large-scale testing first to identify potential false negatives, which could be small. Nonetheless, more extensive studies should be conducted to corroborate these findings.

## Conclusion

In this study, we have shown the advantage of combining serological methods with RT-PCR for SARS-CoV-2 detection as a valuable tool for precise diagnosis. Since some suspected COVID-19 cases were tested negative twice with RT-PCR and seropositive for IgM and IgG SARS-CoV-2 specific antibodies, while expressing COVID-19-related symptoms and have been in close contact with confirmed cases. These results could also provide a strategy to prevent or lead to rapid control of future outbreaks.

##  Supplemental Information

10.7717/peerj.15024/supp-1 Table S1 Characteristics of study participants

10.7717/peerj.15024/supp-2 Table S2 Factors associated with study participants

10.7717/peerj.15024/supp-3 Data S1 Data for tables and figures

## Abbreviations

SARS-CoV-2: Severe acute respiratory syndrome coronavirus-2
COVID-19: Coronavirus disease 2019
RT-PCR: Reverse transcription-polymerase chain reaction
HCWs: Healthcare workers
ELISA: Enzyme-linked immunosorbent assay
RT: Room temperature
TMB: 3, 3′, 5, 5′-tetramethylbenzidine
DMEM: Dulbecco’s modified essential medium
FBS: Fetal bovine serum
VSV: Vesicular stomatitis virus
rVSV- ΔG/SARS-2-Spike: Recombinant vesicular stomatitis virus (VSV) bearing SARS-CoV-2 spike protein
ODs: Optical densities

## Additional Information and Declarations

### Data Availability

The following information was supplied regarding data availability: The raw measurements are available in the Supplementary File.
