# Supplementary material for: The importance of combining serological testing with RT-PCR assays for efficient detection of COVID-19 and higher diagnostic accuracy
Source: PeerJ. 2023 Apr 11;11:e15024. doi: 10.7717/peerj.15024 (PMC10103696; doi:10.7717/peerj.15024)
Supplement: Table S1 [file peerj-11-15024-s001.docx]

**Supplementary Tables**

**Table S1. Characteristics of study participants**

| **Characteristic** | **Category** | **Number of participants**  **n (%)** |
| --- | --- | --- |
| **overall** |  | **9** |
| **Gender^a^** | Male  Female | 1 (11.1%)  8 (88.8%) |
| **Age^b^** | 37.2 ± 9.78 | |
| **Professional category^a^** | HCW | 9 (100%) |
| **In contact with patients^a^** | Yes  No | 7 (77.7%)  2 (22.22%) |
| **In contact with COVID-19 confirmed cases^a^** | Yes | 9 (100%) |
| **Diagnosed previously with COVID-19^a^** | No | 9 (100%) |
| ^a^ n (column percentage).  ^b^Mean ± SD | | |
