# Supplementary material for: The importance of combining serological testing with RT-PCR assays for efficient detection of COVID-19 and higher diagnostic accuracy
Source: PeerJ. 2023 Apr 11;11:e15024. doi: 10.7717/peerj.15024 (PMC10103696; doi:10.7717/peerj.15024)
Supplement: Table S2 [file peerj-11-15024-s002.docx]

**Supplementary Tables**

**Table S2 Factors associated with study participants**

| **Characteristic** | **Category** | **Seropositive**  **n (%)** | ***P-value*** |
| --- | --- | --- | --- |
|  |  |  |  |
| **Gender^a^** | Male  Female | 1 (11.1%)  8 (88.8%) | 0.0034 |
| **Age^b^** | 30.6 ± 4.56  45.5 ± 7.93 | 5 (55.5%)  4 (44.4%) | >0.9999 |
| **Expressed Symptoms** | Yes  No | 7 (77.7%)  2 (22.2%) | 0.056 |
| **Swab type^a^** | Nasal  Oral | 5 (55.5%)  4 (44.4%) | >0.9999 |
| **In-contact with COVID-19 confirmed cases^a^** | Yes | 9 (100%) | <0.0001 |
| a n (column percentage).  b Mean ± SD  *P-value* calculated by Chi-square test | | | |
